# A combination of metabolic resistance and high frequency of the 1014F *kdr* mutation is driving pyrethroid resistance in *Anopheles coluzzii* population from Guinea savanna of Cameroon

**DOI:** 10.1186/s13071-019-3523-7

**Published:** 2019-05-27

**Authors:** Amen N. Fadel, Sulaiman S. Ibrahim, Magellan Tchouakui, Ebai Terence, Murielle J. Wondji, Micareme Tchoupo, Samuel Wanji, Charles S. Wondji

**Affiliations:** 1 LSTM Research Unit, Centre for Research in Infectious Diseases (CRID), P.O. Box 13591, Yaoundé, Cameroon; 2 Department of Microbiology and Parasitology, University of Buea, P.O. Box 63, Buea, Cameroon; 3 Vector Biology Department, Liverpool School of Tropical Medicine (LSTM), Liverpool, L3 5QA UK; 4 Department of Biochemistry, Bayero University, PMB 3011, Kano, Nigeria

**Keywords:** *Anopheles coluzzii*, Deltamethrin, Permethrin, DDT, Metabolic resistance, *kdr*, Cameroon

## Abstract

**Background:**

The scale-up in the distribution of long-lasting insecticidal nets (LLINs) and indoor residual spraying has significantly reduced malaria burden and mortality. However, insecticide resistance, among other factors, is responsible for a recent rebound in malaria transmission in 2015–2016, threatening the progress so far made. As a contribution towards understanding patterns of resistance and its mechanism in the field we characterized a population of *Anopheles gambiae* (*s.l*.) from Gounougou, a Guinea savanna of north/central Cameroon.

**Results:**

Indoor collection conducted in September 2017 identified *Anopheles coluzzii* and *Anopheles arabiensis* as the unique *Anopheles* vector species, with abundances of 83 and 17%, respectively. Analysis of infection with TaqMan assays using heads/thoraces of indoor collected females of *An. coluzzii* revealed a low *Plasmodium falciparum* parasite rate of 4.7%. Bioassays conducted with female *An. coluzzii* revealed extreme resistance, with low mortalities of only 3.75 ± 1.25%, 3.03 ± 1.59% and 1.45 ± 1.45%, respectively, for permethrin, deltamethrin and DDT. In contrast, high susceptibility was obtained with the organophosphates and carbamates, with mortalities in the range of 98–100%. Synergist assays with piperonyl butoxide (PBO) recovered some susceptibility with increased mortality for permethrin to 14.88 ± 8.74%, and for deltamethrin to 32.50 ± 10.51% (~27-fold increase compared to mortalities with deltamethrin alone, *χ*^2^ = 29, *df* = 1, *P* < 0.0001). These correlated with the results of cone bioassays which revealed complete loss of efficacy of Olyset®Net (0% mortality) and PermaNet®2.0 (0% mortality), and the considerable loss of efficacy of Olyset®Plus (mortality of 2 ± 2%), PermaNet®3.0 side panel (mortality of 2 ± 2%) and PermaNet3.0® roof (mortality of 16 ± 5.1%). Time-course bioassays conducted with deltamethrin established a high intensity of resistance, with LT_50_ of 309.09 (95% CI 253.07–393.71, Fiducial), and a resistance ratio of 93.09 compared with the fully susceptible Ngoussou laboratory colony. TaqMan genotyping revealed a high frequency of the 1014F allele (65.25%) in the *An. coluzzii* populations. Sequencing of a fragment of the voltage-gated sodium channel identified a single *An. arabiensis* female harbouring the 1014S *kdr* mutation.

**Conclusions:**

This finding of high pyrethroid and DDT resistance in *An. coluzzii* from north-central Cameroon is a major obstacle to malaria control using pyrethroid bednets and indoor residual spraying with DDT.

## Background

Malaria is one of the major life-threatening diseases in the world, with 435,000 deaths in 2017 [1], of which ~92% occurred in the WHO African region. It is estimated that every two minutes a child dies from this preventable and curable disease [2]. Despite the huge investment in malaria control, conservative estimates suggest that malaria cases increased globally by two million in 2017 compared to 2016 [1, 2]. Insecticide resistance is one of the major obstacles hindering the effectiveness of the core malaria control tools, for example, long-lasting insecticidal nets (LLINs) and indoor residual spraying (IRS) [3, 4].

In Cameroon malaria is endemic, with some variation in the transmission intensity in specific areas, such as highland, Sahel and Guinea savanna [5–7], with *Plasmodium falciparum* as the most common species [8–11]. Malaria transmission in the north of Cameroon is characterized by seasonal patterns linked to rainy season, which covers the period from August to October [6]. In northern Cameroon, the main malaria vectors belong to the *An. gambiae* complex, *Anopheles funestus* group, *Anopheles pharoensis* [5, 12–15] and recently *Anopheles rufipes* [16, 17]. Members of the *An. gambiae* complex largely distributed in northern Cameroon are *An. arabiensis*, *An. coluzzii* and *An. gambiae* (*s.s.*) [18, 19]. It has been shown in studies conducted across four ecogeographical zones of Cameroon, from the northern savanna region to southern forested regions, that in most urban settings *An. coluzzii* (the M molecular form) densities are greater than those of *An. gambiae* (*s.s*.) (the S form), which is more prevalent in peri-urban and rural settings [20, 21].

Intense use of the pyrethroid insecticides in northern Cameroon for crop protection by farmers [13, 15] and malaria vector control through large scale insecticide-treated nets (ITNs) and LLIN distribution programmes [22] could possibly have placed selective pressure on the malaria vectors [4] to develop more resistance. Starting from 2017, Cameroon has scaled-up the distribution of ITNs and IRS in the northern part of the country [23], including Gounogou. This, in addition to the use of organochlorides, carbamates and pyrethroids in Gounougou cotton farms [13, 19] could have resulted in increased resistance to public heath insecticides.

The two major resistance mechanisms, metabolic and *kdr*-based, have been increasingly reported in *An. gambiae* complex and only metabolic mechanism in *An. funestus* (*s.s.*) from northern Cameroon [19]. Metabolic-based resistance is the major mechanism in the north tropical regions of Cameroon, with the temporal distribution of the DDT/pyrethroid 119F-GSTe2 resistant allele detected in *An. funestus* (*s.s.*) [15]. Additionally, overexpressed cytochrome P450s are implicated in conferring increased tolerance to pyrethroids in *An. funestus* and *An. gambiae* (*s.l*.), e.g. *CYP4G16* has been shown to be involved in deltamethrin tolerance in *An. arabiensis* mosquito populations from northern Cameroon [15, 24].

The presence of the 1014F and 1014S *kdr* mutations in *An. coluzzii* and *An. gambiae* populations from Cameroon was first established by Etang et al. [25]. These mutations have since then spread across the country, from the southern wet forest to the northern dry savanna [19]. Various studies have revealed a widespread distribution of the *kdr* mutations in pyrethroid and DDT resistant populations of *An. gambiae* (*s.l.*) in different localities in northern Cameroon, including Gounougou [13, 14, 26–28].

Among the two major malaria vector species involved in transmission in Gounougou, only *An. funestus* has been sufficiently characterized, whereas little is known about *An. gambiae* complex. Although vector control is highly prioritized in the Cameroonian national strategic plan for malaria control, a lack of evidence on the molecular basis of metabolic resistance in *An. gambiae* (*s.l.*) from northern Cameroon is hindering the implementation of evidence-based control tools and resistance management. To support malaria vector control in northern Cameroon, we characterized a population of *An. gambiae* (*s.l*.) from Gounougou, a village in the Guinea savanna of Lagdo. *An. coluzzii* and *An. arabiensis* were found sympatric at Gounougou, with the former in higher proportion. The *An. coluzzii* exhibited high resistance to the pyrethroids and DDT. A high frequency of the 1014F *kdr* mutation was detected in *An. coluzzii*, while the mutation was absent in *An. arabiensis*. In contrast, a single *An. arabiensis* female was found harbouring the 1014S *kdr* allele.

## Methods

### Sampling site and mosquito collection

**Fig. 1:**
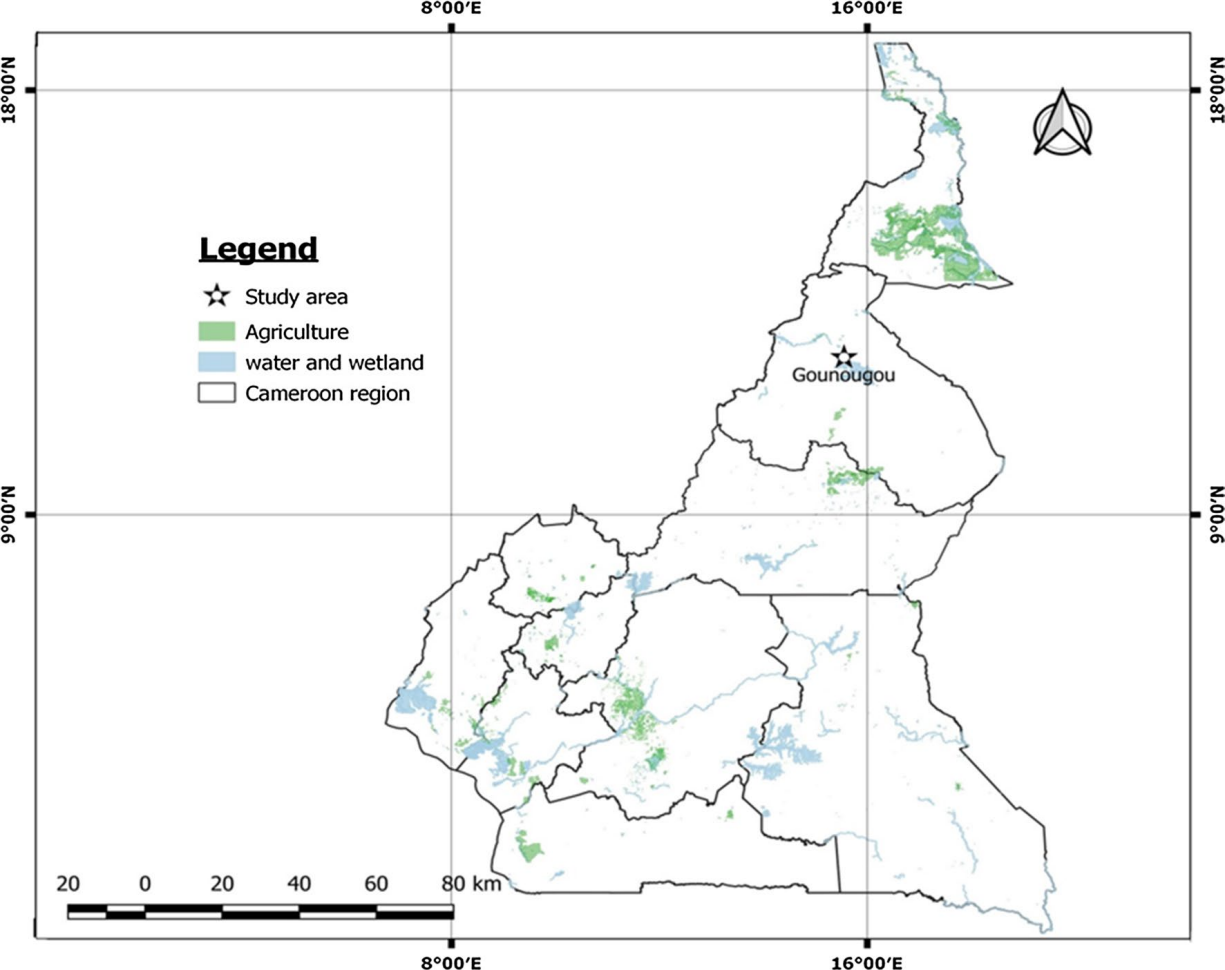
A map of the sampling locality, Gounougou, northern Cameroon

Mosquitoes were collected at Gounougou (9°03′00″N, 13°43′59″E), a town located at Lagdo, in northern Cameroon (Fig. 1). This locality lies along the River Benoué, in an area with cotton farming and rice cultivation, made possible by the presence of a hydroelectric dam. Farmers at Gounougou have a long tradition of using DDT and pyrethroid insecticides [26]. The climate of Gounougou is characterized by a short rainy season from May to September (mean annual rainfall of 900 to 1000 mm) and a long dry season from October to April. Between 2011 and 2016, almost all households at Gounougou had been provided with LLINs by the Cameroon National Malaria Control Programme [19]. PermaNet®2.0 containing deltamethrin and Olyset®Net containing permethrin were distributed at Gounougou with a coverage of ~80% in 2016 and 85% in 2018.

Blood-fed female *Anopheles* mosquitoes resting indoors were collected between 06:00 and 08:00 h in August 2017. This was done using Prokopack electrical aspirators (John W. Hook, Gainesville, FL, USA). Mosquitoes were kept in paper cups in cooling boxes during transport to the insectary at OCEAC, Cameroon. The previously described forced-egg laying method [29] was used to generate F_1_ progenies in 1.5 ml microcentrifuge tubes containing a slightly wet filter paper. Eggs were stored at room temperature for up to 2 days, and then transferred into small paper cups to hatch. Larvae were transferred to bowls containing water for rearing. Mosquitoes were maintained under standard insectary conditions, with a temperature 25 °C, relative humidity of 70–80% and 12:00 h light:dark cycles. Emerged adults were maintained on 10% sucrose in cages, and randomly mixed for insecticide bioassays.

### Mosquito species identification to the species level

A total of 209 female *Anopheles* mosquitoes were collected at Gounougou, out of which 202 (96.65%) were identified morphologically according to the keys of Gillies & de Meillon [30] and Gillies & Coetzee [31]. These comprised 195 *An. gambiae* (*s.l.*) (96.5%) and 7 *An. rufipes* (3.5%). Only 123 F_0_ *An. gambiae* (*s.l.*) which laid eggs successfully with positive hatching were identified to the species level using PCR. It was the larvae of these PCR-identified females which were pooled to generate F_1_ progenies. The Livak method [32] was used to extract DNA and identification to species level was carried out using SINE200 PCR for *An. gambiae* complex [33].

### Estimation of *Plasmodium* spp. infection rates

The sporozoite infection rate was determined using heads/thoraces of 106 blood-fed F_0_ females collected indoors. These females were allowed to lay eggs before being dissected. The TaqMan assay was carried out as previously established [34] using the real-time PCR MX 3005 (Agilent, Santa Clara, CA, USA). One microliter of DNA was used as a template in a 3-step PCR with denaturation at 95 °C for 10 min, followed by 40 cycles of 95 °C for 15 s and 60 °C for 1 min. The primers PlasF (5′-GCT TAG TTA CGA TTA ATA GGA GTA GCT TG-3′) and PlasR (5′-GAA AAT CTA AGA ATT TCA CCT CTG ACA-3′) were used together with two probes labelled with flurorophores FAM (Falcip+ 5′-TCT GAA TAC GAA TGT C-3′) to detect *P. falciparum*, and HEX (OVM+ 5′-CTG AAT ACA AAT GCC-3′) to detect *P. ovale*, *P. vivax* and *P. malariae*. Two *P. falciparum* samples and a mix of *P. ovale*, *P. vivax* and *P. malariae* were used as positive controls. A nested PCR of Snounou et al. [35] was performed for all the positive samples to validate the TaqMan assay results.

### Insecticide susceptibility bioassays

To establish resistance profile of the *An. gambiae* (*s.l.*) populations, various insecticides used in public health control of malaria vectors were tested in bioassays following the protocol of the World Health Organization [36]. Two- to four-day-old, unfed F_1_ and F_2_ female *An. coluzzii* were exposed for 1 h to diagnostic doses of the following insecticides: the type I pyrethroid permethrin (0.75%), and the type II pyrethroid deltamethrin (0.05%); the organochlorine, DDT (4%); the carbamates, bendiocarb (0.01%) and propoxur (0.1%); and the organophosphates, malathion (5%) and fenitrothion (5%). Four replicates of approximately 20–25 mosquitoes per tube (except for deltamethrin, F_1_ tested with three replicates) were exposed to impregnated papers for 1 h and then transferred to holding tubes. Mosquitoes were supplied with 10% sucrose and mortalities were recorded at 24 h after exposure. The control comprised 25 mosquitoes exposed to untreated papers. Populations were considered susceptible to an insecticide where mortality was > 98%, suspected to be resistant (moderately resistant) where mortality was between 90–98%, and resistant where mortality was found to be < 90% [36].

### Synergist bioassay with piperonyl butoxide (PBO)

To identify the possible enzyme systems involved in pyrethroids and/or DDT resistance synergist bioassays were conducted. Due to a low number of mosquitoes, the F_1_ progenies were allowed to mate and were blood-fed to generate F_2_ populations. Two- to four-day-old F_2_ females were first exposed to 4% PBO for 1 h, followed by exposure to either 0.75% permethrin, 0.05% deltamethrin or 4% DDT for 1 h. Mortality was recorded 24 h after exposure and the differences in mortalities between synergized and non-synergized experiments were compared using a Chi-square test of significance.

### Test of bednet efficacy using a cone assay

Cone bioassays were conducted according to the WHO procedure [36] using unfed, 3- to 4-day-old F_2_ females of *An. coluzzii*. Five replicates of ten mosquitoes were placed in plastic cones attached to four standard insecticide bednets: the Olyset®Net (containing 2% permethrin), OlysetPlus®Net (containing 2% permethrin combined with 1% of the synergist PBO), PermaNet®2.0 (containing 1.4–1.8 g/kg ± 25% deltamethrin), and PermaNet®3.0 [both the side panel (containing 2.1–2.8 g/kg ± 25% deltamethrin) and the roof (containing 4.0 g/kg ± 25% deltamethrin, combined with 25g/kg ± 25% of PBO)]. Fresh, unused PermaNets and OlysetNets were respectively provided by Vestergaard, Lausanne, Switzerland and Sumitomo Chemical Plc, London, UK. Three replicates of ten mosquitoes were exposed to an untreated net as control. For each test, the exposure time was 3 min and mosquitoes were immediately transferred to paper cups and supplied with 10% sucrose. Mosquitoes that were knocked down were recorded after 60 min and mortality recorded 24 h after exposure.

### Establishment of resistance intensity

To assess the strength of resistance, a time course bioassay was conducted using deltamethrin with F_2_ *An. coluzzii* populations. The protocol followed the procedure as outlined for the conventional bioassays only that exposure time was varied, using exposures of 60, 90, 120, 180 and 240 min. The time required to kill 50% of the experimental mosquitoes (LT_50_) was calculated using the glm function of MASS package (R v.3.5.0). The Ngoussou females were also exposed to deltamethrin in a time-course bioassay at 2.5, 5, 10, 15, 30, 45 and 60 min, and LT_50_ was calculated. Resistance intensity was estimated from the ratio of the LT_50_ of the Gounougou populations to that of Ngoussou. Measurement of resistance intensity using 5× and 10× insecticide concentrations, as recommended by the WHO, was not undertaken due to unavailability of the papers.

### Investigation of the role of the 1014F knockdown resistance mutation in pyrethroid/DDT resistance

To establish the frequency of *kdr* mutations in field caught *An. gambiae* (*s.l.*) populations, a TaqMan assay was performed to genotype the L1014F *kdr* mutation according to the procedure of Bass et al. [37]. Ten microliters containing 1× Sensimix (Bioline, TN, USA), 80× primer/probe mix and 1 µl of template DNA were used. The primers *kdr*_F (5′-CAT TTT TCT TGG CCA CTG TAG TGA T-3′) and *kdr*_R (5′-CGA TCT TGG TCC ATG TTA ATT TGC A-3′) were used without modififcation. Probes were labelled with two specific fluorophores, FAM and HEX: FAM to detect the resistant allele [(5′-ACG ACA AAA TTT C-3′ for 1014F *kdr*), (5′-ACG ACT GAA TTT C-3′ for 1014S *kdr*)] and HEX (5′-CTT ACG ACT AAA TTT C-3′) to detect the susceptible allele. The assay was performed on an Agilent MX3005 real-time PCR machine with cycling conditions of 95 °C for 10 min, followed by 40 cycles of 95 °C for 15 s and 60 °C for 1 min.

To assess the genetic diversity and detect potential signatures of selection acting on the voltage-gated sodium channel, a portion of this gene spanning exon 20 was sequenced in 15 *An. coluzzii* and 14 *An. arabiensis* females. Initial fragment amplification was carried out using primers *kdr*CL-F (5′-AAA TGT CTC GCC CAA ATC AG-3′) and *kdr*CL-R (5′-GCA CCT GCA AAA CAA TGT CA-3′) described previously [38]. The 15 µl PCR mixture comprised 1.5 µl of 10× Buffer A (Kapa Biosystems, Wilmington, MA, USA), 0.75 µl of 25 mM MgCl_2_, 0.12 µl of 25 mM dNTPs, 0.12 µl of Kapa Taq DNA polymerase, 0.51 µl each of the forward and reverse primer, 10.49 µl of double distilled water (ddH_2_O) and 1 µl of genomic DNA template. Cycling condition were as follows: 95 °C for 5min, followed by 35 cycles of 94 °C for 30 s, 57 °C for 30 s and 72 °C for 45 s, followed by a final extension step at 72 °C for 10 min. PCR products were cleaned up using Exonuclease I (Exo I) and Shrimp Alkaline Phosphate (Exo-SAP protocol) according to the protocol of New England Biolabs (NEB, MA, USA). Purified PCR products were sequenced on both strands using the above primers.

Sequences were corrected manually using BioEdit v.7.2.1 [39] and aligned with ClustalW. Analysis of genetic parameters of polymorphism was carried out using theDnaSP v.5.10 [40]. Different sequences/haplotypes were compared by constructing a maximum likelihood phylogenetic tree, using MEGA v.7.0 [41].

## Results

### Mosquito species composition

All mosquitoes collected indoors were morphologically identified as *An. gambiae* (*s.l*.) and *An*. *rufipes*. The 123 females which laid eggs were PCR-identified as 83% *An. coluzzii* (102 females) and 17% *An. arabiensis* (21 females).

### Sporozoite infection rate

Out of 106 *An. gambiae* (*s.l.*) females analysed by TaqMan assays (85 *An. coluzzii* and 21 of *An. arabiensis*), only 4.7% of the *An. coluzzii* (4/106) were infected with *P. falciparum*. These four positive samples were validated by nested PCR. None of the *An. arabiensis* mosquitoes were infected.

### Insecticide resistance status of *An. coluzzii* populations

**Fig. 2:**
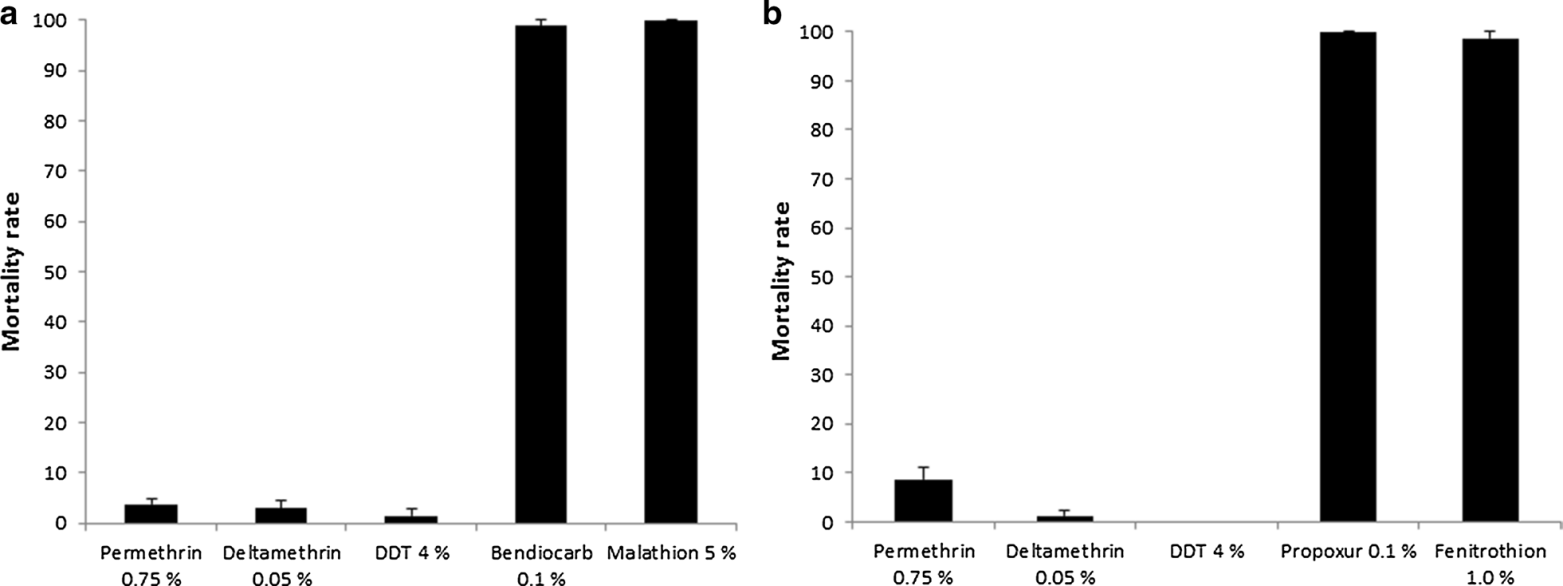
Results of WHO insecticides susceptibility test. **a** Susceptibility profile of female Gounougou *An. coluzzii* (F_1_ population) following exposure to various public health insecticides. **b** Susceptibility profile of Gounougou female *An. coluzzii* (F_2_ population). Error bars represent standard error of the mean

Only *An. coluzzii* populations (F_1_ and F_2_ generations) were used for bioassays. *Anopheles arabiensis* were not used due to the low number during collection, and low hatching rate/larvae survival. The *An. coluzzii* populations were highly resistant to pyrethroids, with mortalities on exposure to permethrin of 3.75 ± 1.25% and 8.75 ± 2.23% for the F_1_ and F_2_ progenies, respectively (Fig. 2a, b). Higher resistance was observed with the type II pyrethroid-deltamethrin, with mortalities for the F_1_ and F_2_ progenies of 3.03 ± 1.59% and 1.19 ± 1.19%, respectively. High resistance was also seen with DDT (mortality of 1.45 ± 1.45% for F_1_ progenies, and no mortality at all with the F_2_ progenies). However, all mosquitoes were susceptible to carbamates, with mortalities for bendiocarb of 98.81 ± 1.19% for the F_1_ progenies, and 100% for propoxur with the F_2_ progenies. The same pattern of susceptibility was obtained with organophosphates, with malathion producing 100% mortality for F_1_ progenies and fenitrothion exhibiting a mortality of 98.75 ± 1.25% for the F_2_ progenies.

### Synergist bioassay for pyrethroid and DDT resistance

**Fig. 3:**
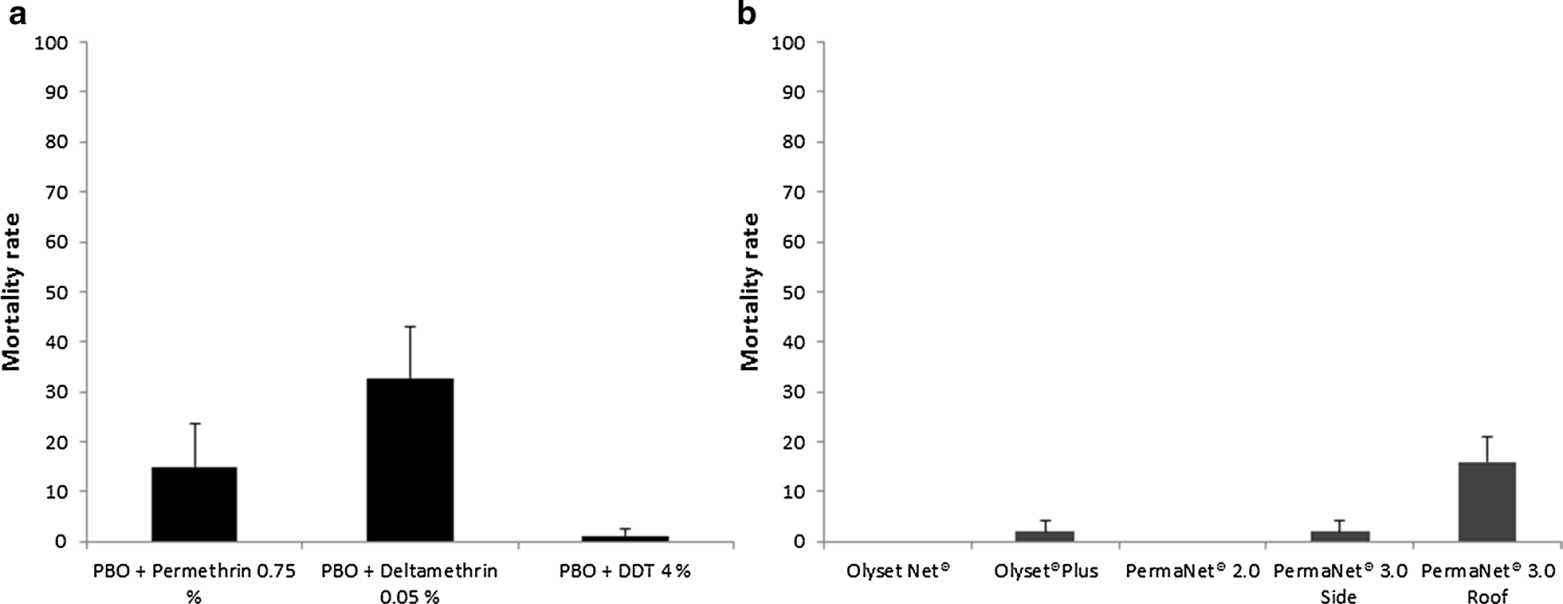
Results of synergist bioassays and cone bioassays. **a** Effect of pre-exposure to piperonyl butoxide (PBO) on mortality in female F_2_ *An. coluzzii.* **b** Effect of exposure to various LLINs on mortality in female *An. coluzzii* populations. Error bars represent standard error of the mean

To investigate the possible role of the cytochrome P450s in the observed pyrethroid and DDT resistance, the F_2_ *An. coluzzii* were pre-exposed to PBO followed by permethrin, deltamethrin and DDT separately. Compared to results from conventional bioassays above (mortality of 8.75 ± 2.23%), no significant recovery of susceptibility was observed for permethrin, with mortality increasing to only 14.88 ± 8.74% following pre-exposure to PBO (Fig. 3a). A significant increase in mortality was observed in mosquitoes exposed to PBO and deltamethrin (mortality of 32.50 ± 10.51%) compared with those exposed to deltamethrin alone (*χ*^2^ = 29, *df* = 1, *P* < 0.0001). This is, on average, a 27-fold increase. For DDT, there was no major change in mortality following PBO pre-exposure (1.19 ± 1.19%). No mortality was recorded in control mosquitoes exposed to the PBO alone.

### Determination of bednet efficacy

To evaluate efficacy of the long-lasting insecticidal nets, cone bioassays were carried out. Initially, a 100% mortality was observed with the fully susceptible *An. coluzzii* (Ngoussou laboratory colony) with PermaNet®2.0, and a high mortality of 88 ± 3.74% with Olyset®Net. On testing the Gounougou population (F_2_ *An. coluzzii*), an extensive loss of efficacy of Olyset®Net and Permanat®2.0 was observed, with 0% mortality 24 h after exposure (Fig. 3b). Low mortalities were also obtained with the side panels of PermaNet®3.0 (2 ± 2%) and Olyset®Plus (2 ± 2%) with the F_2_ populations. Exposure to the roof of PermaNet®3.0 increased the mortalities to 16 ± 5.1% indicating a limited role of cytochrome P450s in the deltamethrin resistance. No mortality was obtained with the F_2_ control populations exposed to untreated bednets.

### Intensity of pyrethroid resistance in *An. coluzzii* populations

**Fig. 4:**
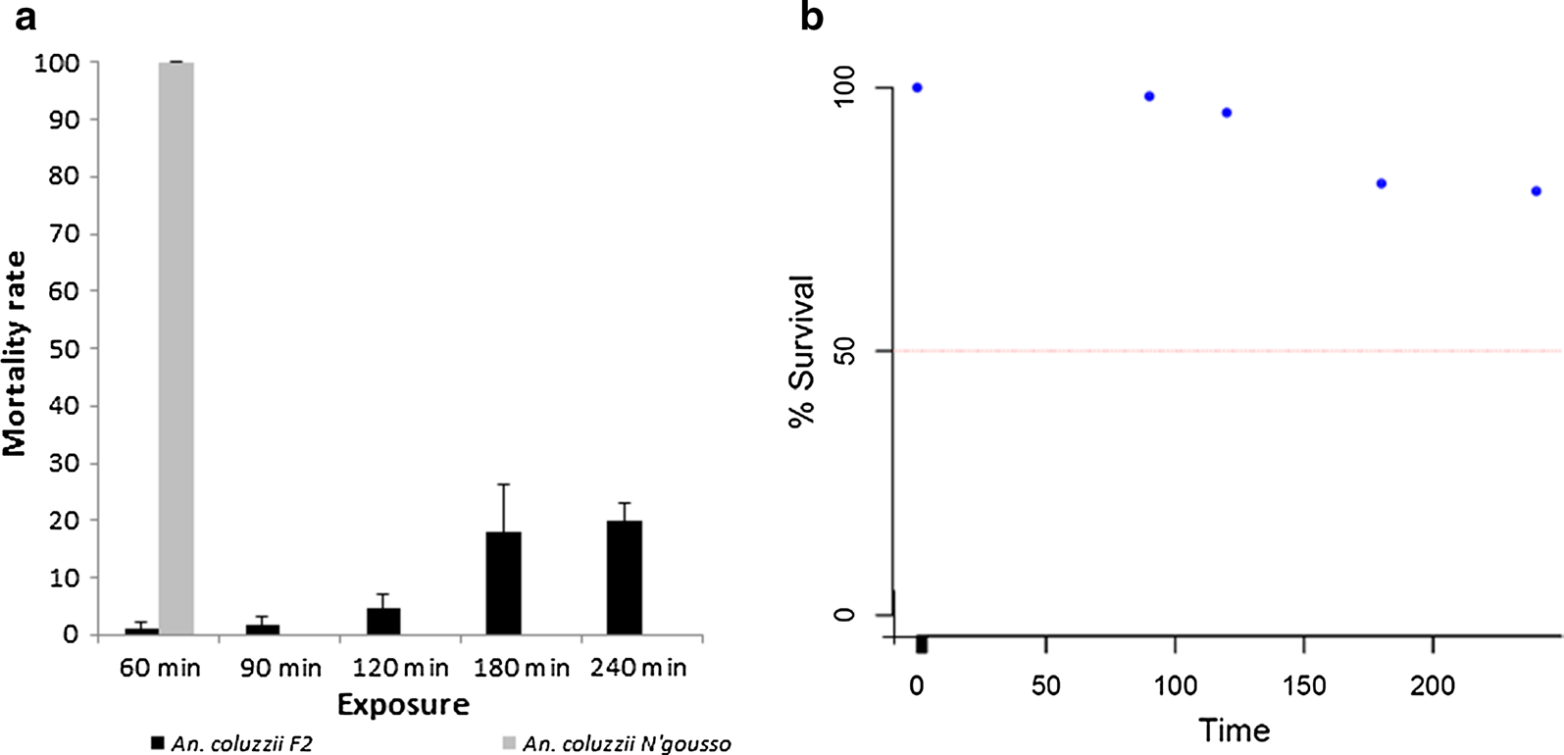
Results of the time-course bioassay with deltamethrin. **a** Susceptibility profile of female *An. coluzzii* population in Gounougou and *An. coluzzii* N’gousso strain to deltamethrin (0.05%) at different time points. Error bars represent standard error of the mean. **b** Probit plot for estimation of resistance intensity in *An. coluzzii* populations

Time course bioassays confirmed intense deltamethrin resistance in the Gounougou *An. coluzzii* (Fig. 4a). Mortalities of less than 5% were obtained after 2 h of exposure and less than 20% after 4 h. This led to a very high LT_50_, estimated as 309.09 (95% CI: 253.07–393.71, Fiducial) (Fig. 4b). The LT_50_ for Ngoussou colony was calculated as 3.32 min (CI: 2.67–3.97) from a previous study [42]. Thus, a resistance intensity for the Gounougou population compared with Ngoussou was calculated as 93.1.

### Presence of the knockdown resistance mutation

To establish the frequency of 1014F *kdr* mutation in the Gounougou population, a TaqMan genotyping was carried out using DNA samples extracted from 59 F_0_ *An. coluzzii* and 18 F_0_ *An. arabiensis*. The 1014F *kdr* mutation was only found in the *An. coluzzii* population, at a high frequency of 65.25%. The frequency of this *kdr* was marginally higher in the heterozygote individuals, at 45.7% (27/59), compared to the homozygous resistant individuals (42.37%, 25/59). The distribution of *kdr* genotype is shown in Table 1. The susceptible allele 1014L was detected in both species, with 7 *An. coluzzii* homozygous susceptible (7/59, 11.87%), and all the *An. arabiensis* 100% homozygote susceptible (18/18). The L1014S *kdr* mutation was not investigated using TaqMan assay.

**Table 1:** Genotypes and allele frequency of the 1014F *kdr* mutation in the Gounougou *An. coluzzii* population

Population	Genotype					Allele	
	SS	RR	RS	Total	2*N*	f(S)	f(R)
*An. coluzzii*	7	25	27	59	118	0.3475	0.6525
*An. arabiensis*	18	0	0	18	36	1	0

*Abbreviations*: RR, homozygous resistant; RS, heterozygous resistant; SS, homozygous susceptible

### Polymorphism analysis of the *An. coluzzii* voltage-gated sodium channel (VGSC)

**Fig. 5:**
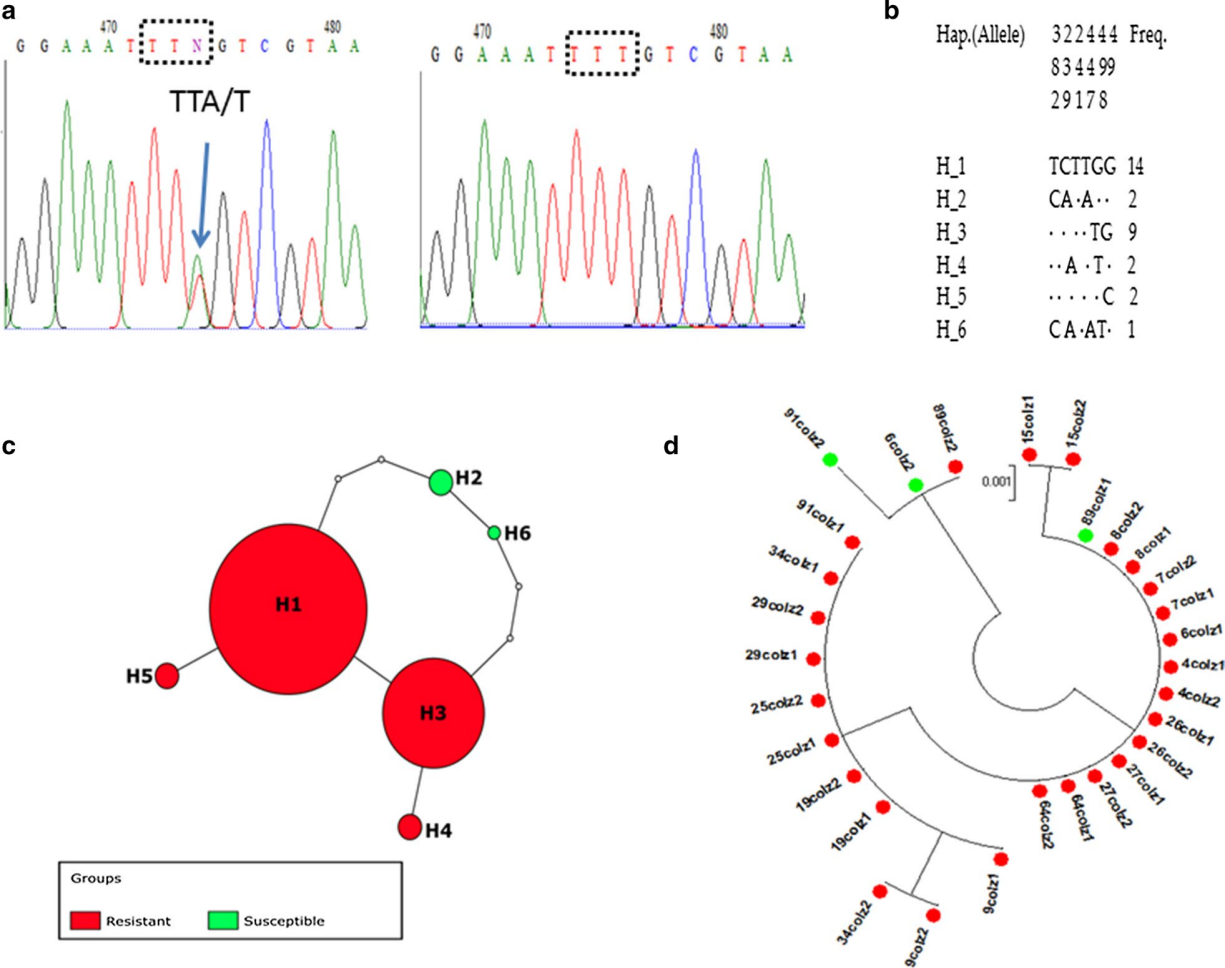
Genetic diversity of fragment of *VGSC* spanning exon 20 in *An. coluzzii*. **a** Sequencing traces showing the polymorphic position for the 1014F *kdr* mutation. **b** Haplotype diversity patterns of the 498-bp fragment of VGSC. **c** TCS and tcsBU haplotype network showing a low polymorphism of exon 20. **d** Phylogenetic tree analysis of the VGSC fragment. Green dots represent the susceptible haplotypes and the red dots the resistant haplotypes

Analysis of 498-bp fragments of the VGSC spanning the 1014 codon from 15 *An. coluzii* indicated the occurrence of six distinct haplotypes in Gounougou females. The genetic diversity parameters of fragment of the VGSC gene are provided in Table 2. Overall, six polymorphic sites were present in the sequenced fragment with a haplotype diversity of 0.701. Four of six haplotypes (H1, H2, H4 and H5) had 1014F resistant alleles at high frequency of 90% (27/30) while the two remaining ones had 1014L susceptible alleles at lower frequency of 10% (3/30). Among these, H1 and H3 were the two major haplotypes containing the 1014F resistant allele at high frequency of 46.66% (14/30) and 30% (9/30), respectively (Fig. 5a-c). The neutrality test of Tajima’s D had a negative value for *An. coluzzii* but it was not significant.

**Table 2:** Summary statistics for polymorphism and diversity in the voltage-gated sodium channel gene of Gounougou *An. coluzzii* and *An. arabiensis*

Species	Gene	*n*	S	h	Hd	π	TajimaD	FuLiD*	FuLiF*
*An. coluzzii*	Exon 20	30	6	6	0.701	0.00381	− 0.384^ns^	1.2143^ns^	0.8598^ns^
*An. arabiensis*	Exon 20	28	6	5	0.27	0.00108	− 1.9719^ns^	− 2.5946^ns^	− 2.8039^ns^

*Abbreviations*: *n*, number of sequences; S, number of polymorphic sites; h, haplotype; Hd, haplotype diversity; π, nucleotide diversity; TajimaD, Tajima’s D statistic; FuLiD*, Fu and Li’s D* statistic; FuLiF*, Fu and Li’s F* statistic; ns, not significant

Phylogenetic analysis of the above six haplotypes using maximum likelihood indicated that there were two distinct haplotype groups, with one carrying the 1014F resistant allele and the other containing the 1014L susceptible allele (Fig. 5d). The comparison of the Gounougou haplotypes with four *kdr* bearing haplotypes previously detected across Africa revealed that only the second major haplotype H3 from Gounougou belongs to the H1-1014F resistant haplotype, predominant in West/Central Africa [38]. Haplotype network tree analysis showed that haplotypes H3, H4 and H5 are separated by 1 or 2 mutational steps from the ancestor haplotype H1 which also belonged to the resistant allele (Fig. 5d). This observation suggests an independent occurrence of the 1014F haplotype in Gounougou, potentially from local selection. Haplotypes H2 and H6 carrying the 1014L susceptible allele are the most isolated to the predominant haplotype H1 with 3 and 4 mutational steps, respectively.

**Fig. 6:**
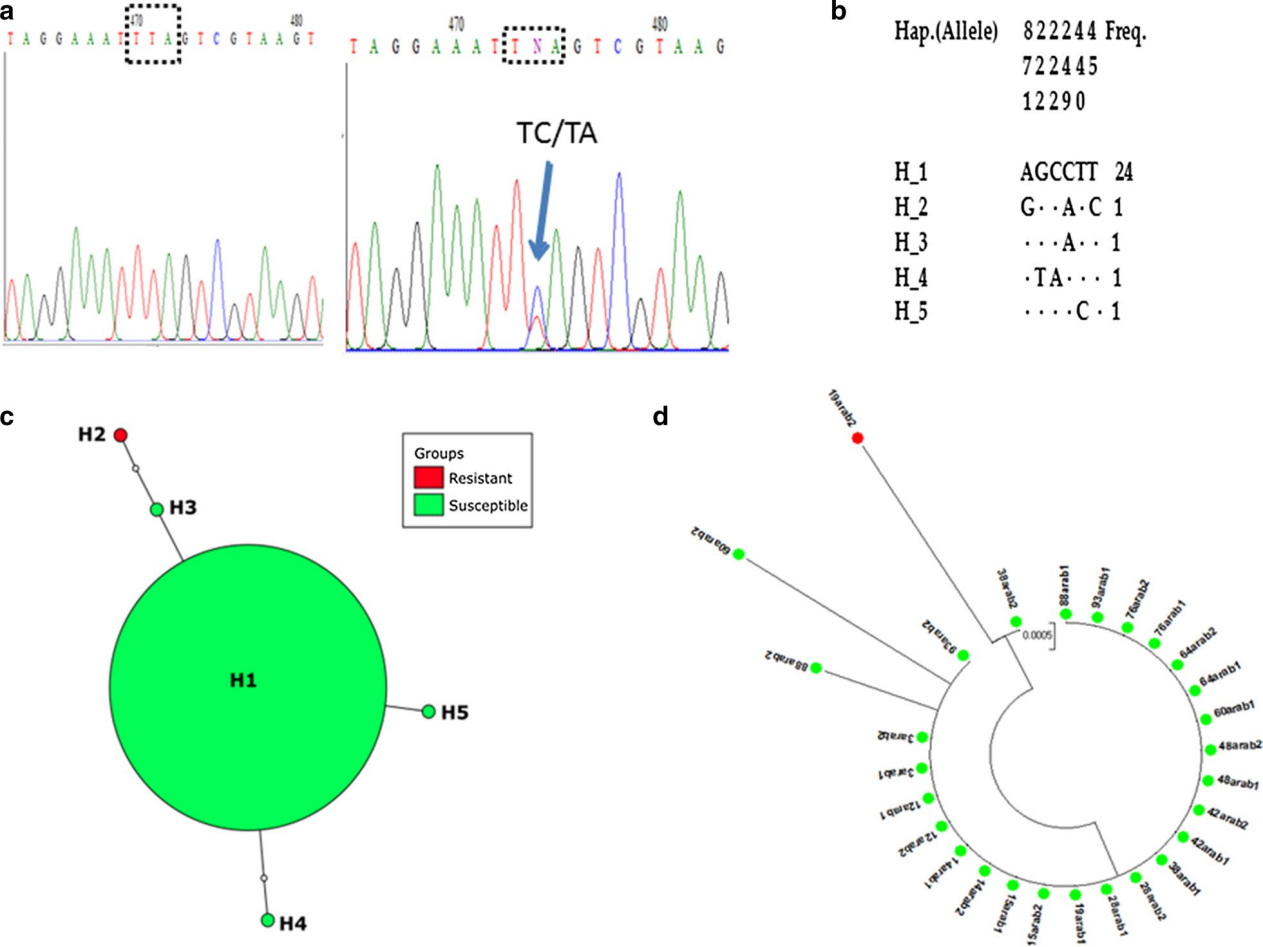
Genetic diversity of fragment of *VGSC* spanning exon 20 in *An. arabiensis*. **a** Sequencing traces showing the polymorphic position 441 generating the 1014S *kdr* mutation. **b** Haplotype diversity patterns of the 460-bp fragment in Gounougou. **c** TCS and tcsBU haplotype network showing a low polymorphism in exon 20. **d** Phylogenetic tree analysis of VGSC. Green dots represent the susceptible haplotype, and the lone red dot the resistant haplotype

Analysis of the haplotype network of the *kdr* gene based on L1014F alleles of 460-bp from 14 *An. arabiensis* showed that there are five haplotypes in total (Fig. 6d), with a very low polymorphism and low haplotype diversity, of only 0.27 (Table 2). Tajima’s neutrality test had negative values, but they were not significant (Table 2). Four of the haplotypes (H1, H3, H4 and H5) contained the 1014L susceptible allele, and surprisingly one haplotype harbored the 1014S resistant allele (Fig. 6d), leading to a low 1014S *kdr* frequency of 3.57% (1/28). Haplotype H1 was the only major haplotype carrying the 1014L susceptible allele at a high frequency of 85.71% (24/28) among the identified haplotypes (Fig. 6a–c). Genetic parameters for the analysis of this fragment of VGSC are summarized in Table 2.

Phylogenetic analysis also established the presence of three haplotypes H3, H4 and H5 carrying 1014L susceptible allele, derived from single or two mutational steps from the predominant haplotype H1 (Fig. 6d). Haplotype H2 containing the 1014S resistant allele is the most isolated to the major haplotype H1 with three mutational steps.

## Discussion

### Composition of the local *An. gambiae* (*s.l.*) populations and their role in malaria transmission

In this study we established the role of the major malaria vector *An. coluzzii* from northern Cameroon in malaria transmission and characterized its insecticide resistance profile. *Anopheles coluzzii* and *An. arabiensis* were the only members of *An. gambiae* species complex obtained at Gounougou. *Anopheles coluzzii* was the most prevalent species from indoor resting collection, whereas *An*. *arabiensis* was found at low frequency. It has been reported that at the height of the rainy season (August) *An. coluzzii* is usually the most dominant species, while in the dry season *An. gambiae* (*s.s.*) and *An. funestus* are the most dominant [15, 26, 43]. The findings of a higher proportion of *An. coluzzii* indoors might perhaps be explained from observations of the more synanthropic nature and endophilic tendency of this species compared to *An*. *arabiensis*, which is known to exhibit more exophilic behavior [44]. The predominance of *An*. *coluzzii* in Gounougou, in the rainy season, is in-line with recent observations across Sudan/Guinean savanna in West and Central Africa, e.g. as observed in Sudan savanna in Auyo, Nigeria [45], in Guinea savannah of Kome, southern Chad [46], at the village of Goden in Sudanese-savanna of Burkina Faso [47] and in Alibori, northern Benin [48].

*Plasmodium falciparum* was the only malaria parasite species detected, at a low rate, in *An. coluzzii*, suggesting low malaria transmission at this site. However, this infection rate is higher than that obtained in 2014 in *An. coluzzii* from Tiko, South-West region of Cameroon [7], but lower than that obtained in 2016 in *An. coluzzii* from Bangui, in Central African Republic [49]. However, a longitudinal survey is needed to establish the contribution of this vector to malaria transmission in this area.

### Pattern of insecticide resistance

A study in 2005 in three sites near Gounougou found that *An. arabiensis* and *An. gambiae* (*s.s.*) (the S molecular form) were resistant to deltamethrin, permethrin and DDT, but completely susceptible to chlorpyrifos-methyl and propoxur [13]. In 2006, *An. arabiensis* and *An. gambiae* (*s.s.*) from the irrigated area of Gounougou were also described to be resistant to lambda-cyhalothrin and deltamethrin, but fully susceptible to fenitrothion and propoxur [26]. Another study in Pitoa, in 2011 described *An. arabiensis* and *gambiae* (*s.s.*) resistant to permethrin, deltamethrin and DDT at increased levels compared to that reported in 2009 [28]. However, in recent years there has been a possible shift in vector composition, with a rise of *An. coluzzii* populations in place of *An. arabiensis*, and predominance of *An. funestus* as observed in recent studies (e.g. [15]) and our work. This shift is also followed with increased pyrethroid resistance and development of resistance towards the non-pyrethroid insecticides [50].

The continuous distribution of LLINs in Cameroon, especially in 2011 and 2016, has resulted in a gradual increase in resistance to pyrethroids/DDT, as reported in Gounougou [19, 51]. The pyrethroids/DDT resistance has become extreme in this major malaria vector. However, this species is found exhibiting full susceptibility to carbamate (bendiocarb and propoxur) and to organophosphate (malathion and fenitrothion), as previously reported in *An. gambiae* (*s.l.*) [26, 27], suggesting that insecticides of these classes will be preferable for potential IRS campaigns. The focus should be shifted to organophosphates (e.g. malathion and fenitrothion) and carbamates (e.g. bendiocarb and propoxur) as alternatives for IRS campaign in Gounougou and neighboring regions sharing a similar ecological setting.

The level pyrethroid and DDT resistance observed in the Gounougou *An. coluzzii* population is higher than resistance in *An. gambiae* (*s.l.*) reported in other locations with similar geographical characteristics: in Kome, southern Chad (permethrin, 26.7% mortality, deltamethrin, 25.4% and DDT, 41.7%); in Auyo, northern Nigeria (deltamethrin, 78.4% mortality and DDT, 44%); and in Djenne, central Mali (deltamethrin, 16% mortality and DDT, 42%) [44, 45, 52]. This increase may be associated with insecticide selective pressure imposed by the introduction of ITNs in Gounougou since 2006, and LLINs since 2015, as observed in the Sudan/Sahelian-zone of Burkina Faso [53]. Another possible explanation of selective pressure at larval level is attributed to the extensive use of insecticide in cotton-growing area communicating with rice farms field throughout the channel of rain runoff [26].

Bioassays were used to quantify the strength of the resistance and make an association of this strength to the effectiveness of current LLINs as vector control tools. The resistance intensity towards deltamethrin was very high in the Gounougou *An. coluzzii* population producing a very high resistance ratio; this was higher than that established for the previously characterized Tororo and Tiofora populations [54, 55]

The finding of susceptibility to carbamate and organophosphate insecticides is generally consistent with the relatively widespread susceptibility of malaria vector populations to these insecticide classes, especially the organophosphates, as observed in West and Central Africa [26, 56–58].

The results of cone assays in this study revealed a considerable loss of efficacy of the LLINs. This finding of reduced efficacy of combination LLINs containing PBO, such as Olyset®Plus is consistent with results obtained recently in Mibellon in the Adamawa region of Cameroon [50], in which a high levels of resistance in *An. funestus* and *An*. *gambiae* populations to pyrethroids were correlated with the loss of efficacy of bednets containing insecticides and/or synergists under laboratory conditions. The PBO-containing bednets, e.g. PermaNet®3.0 usually increase recovery of mortality for most malaria vectors. The low recovery of mortality from exposure to the roof of PermaNet®3.0 is first of its kind in *An. coluzzii* from Gounougou, and in contrast with previous findings in other African countries [54, 59, 60]. This drastic reduction in efficacy of PermaNet®3.0 may be due to selection pressure from massive distribution of combination bednets. The low mortality of *An*. *coluzzii* observed even with PBO combined with permethrin (Olyset®Plus) and deltamethrin (Permanet®3.0 roof) suggests possible alternative mechanisms for efficient detoxification of the PBO itself, or other major non-P450 detoxification genes driving the pyrethroid/DDT resistance in the field.

### Contribution of target site insensitivity mutations to the pyrethroid and DDT resistance

The high resistance to permethrin, deltamethrin and DDT observed in F_1_ and F_2_ progenies of *An*. *coluzzii* could be linked with the high frequency of the L1014F *kdr* mutation (65.25%) established in the F_0_ parents. However, due to high resistance (low number of dead mosquitoes) phenotype-genotype correlation was not assessed. The 1014F *kdr* mutation has been previously identified in *An. arabiensis* and *An. coluzzii* in Gounougou, and in the neighboring locality of Pitoa, in North Cameroon, at lower frequencies [27, 28]. The frequency of the L1014F *kdr* mutation in Gounougou *An. coluzzii* is consistent with previous results in countries bordering Cameroon, e.g. northern Nigeria where a 1014F *kdr* mutation frequency of 83% was reported [45], and in Central African Republic, with 52.3% for the 1014F resistant allele [49].

The low genetic diversity observed in the partial fragment of the VGSC spanning exon 20, harboring the L1014F codon, suggests a restricted polymorphism of VGSC, highlighted by the very low number of haplotypes, as the 1014F allele nears fixation in this population. Our findings are consistent with previous studies reported in Central Africa [49, 60]. The fact that the predominant haplotype H3 matched with that found across Africa (H1-1014F) suggests a gene flow in *An. coluzzii* populations across the region.

## Conclusions

This study provides evidence for drastically high pyrethroid and DDT resistance in *An. coluzzii*, a major malaria vector from Gounougou, northern Cameroon, with the possibility of a complete loss of efficacy of the major malaria control tools, the LLINs and IRS using DDT. The intense resistance is driven possibly by metabolic mechanism and the 1014F *kdr* mutation which was found in high frequency.

## Data Availability

The datasets (DNA sequences) supporting the conclusions of this article are available in the GenBank database under the accession numbers MK548913–MK548927 for *An. coluzzii* sequences and MK548928–MK548941 for *An. arabiensis* sequences.

## Abbreviations

DDT: dichlorodiethyltrichloroethane
DNA: deoxyribonucleic acid
DnaSP: DNA sequence polymorphism
dNTPs: deoxyribonucleoside triphosphates
GSTe2: glutathione S-transferase epsilon 2
IRS: indoor residual spraying
ITN: insecticide-treated net
kdr: knockdown resistance mutation
LLIN: long-lasting insecticidal net
LT: lethal time
MEGA: Molecular Evolutionary Genetics Analysis
PBO: piperonyl butoxide
PCR: polymerase chain reaction
*s.l.*: *sensu lato*
*s.s*.: *sensu stricto*
VGSC: voltage-gated sodium channel
